# Acute neuropharmacological effects of atomoxetine on inhibitory control in ADHD children: A fNIRS study

**DOI:** 10.1016/j.nicl.2014.09.001

**Published:** 2014-09-10

**Authors:** Masako Nagashima, Yukifumi Monden, Ippeita Dan, Haruka Dan, Daisuke Tsuzuki, Tsutomu Mizutani, Yasushi Kyutoku, Yuji Gunji, Daisuke Hirano, Takamichi Taniguchi, Hideo Shimoizumi, Mariko Y. Momoi, Eiju Watanabe, Takanori Yamagata

**Affiliations:** a Department of Pediatrics, Shimotsuke, Japan; b Department of Neurosurgery, Shimotsuke, Japan; c Functional Brain Science Laboratory, Jichi Medical University, 3311-1 Yakushiji, Shimotsuke, Tochigi 329-0498, Japan; d Applied Cognitive Neuroscience Laboratory, Chuo University, 1-13-27 Kasuga, Bunkyo, Tokyo 112-8551, Japan; e Department of Pediatrics, International University of Health and Welfare, 537-3 Iguchi, Nasushiobara, Tochigi 329-2763, Japan; f International University of Health and Welfare, 2600-1 Kitakanemaru, Otawara, Tochigi, Japan; g Rehabilitation Center, International University of Health and Welfare, 2600-1 Kitakanemaru, Otawara, Tochigi 324-8501, Japan

**Keywords:** Cortical hemodynamics, Developmental disorder, Dorsolateral prefrontal cortex, Optical topography, Stop signal task

## Abstract

The object of the current study is to explore the neural substrate for effects of atomoxetine (ATX) on inhibitory control in school-aged children with attention deficit hyperactivity disorder (ADHD) using functional near-infrared spectroscopy (fNIRS). We monitored the oxy-hemoglobin signal changes of sixteen ADHD children (6–14 years old) performing a go/no-go task before and 1.5 h after ATX or placebo administration, in a randomized, double-blind, placebo-controlled, crossover design. Sixteen age- and gender-matched normal controls without ATX administration were also monitored. In the control subjects, the go/no-go task recruited the right inferior and middle prefrontal gyri (IFG/MFG), and this activation was absent in pre-medicated ADHD children. The reduction of right IFG/MFG activation was acutely normalized after ATX administration but not placebo administration in ADHD children. These results are reminiscent of the neuropharmacological effects of methylphenidate to up-regulate reduced right IFG/MFG function in ADHD children during inhibitory tasks. As with methylphenidate, activation in the IFG/MFG could serve as an objective neuro-functional biomarker to indicate the effects of ATX on inhibitory control in ADHD children. This promising technique will enhance early clinical diagnosis and treatment of ADHD in children, especially in those with a hyperactivity/impulsivity phenotype.

## Highlights

• We assessed the effects of atomoxetine administration to ADHD children using fNIRS.
• Normal healthy control subjects recruited the right IFG/MFG during go/no-go tasks.
• Pre-medicated ADHD children exhibited reduced right IFG/MFG activation.
• The activation was acutely normalized by atomoxetine, but not by placebo.
• The right IFG/MFG activation may serve as an objective neuro-functional biomarker.

## 1 Introduction

Attention Deficit Hyperactivity Disorder (ADHD) is one of the most prevalent developmental disorders, affecting between 5 and 9% of school-aged children (Dittmann et al., 2009; Willcutt, 2012). ADHD is associated with a primary impairment in executive controls, including response inhibition and working memory (Barkley, 1997; Dias et al., 2013; Dittmann et al., 2009; Willcutt, 2012). Symptoms of ADHD typically develop during early elementary school years, and, in most cases, progress to a chronic state during adulthood (Drechsler et al., 2005). Because of this, initiating appropriate treatment in youth upon early identification is important in order to confer long-term positive effects. Recommended treatments for ADHD children include both medication and behavioral therapy (Hodgkins et al., 2012).

The non-stimulant drug, atomoxetine (ATX) as well as the stimulant drug, methylphenidate (MPH) have been recommended as primary medications for the improvement of executive function in ADHD patients (Cubillo et al., 2014; Faraone and Buitelaar, 2010; Faraone et al., 2007; Newcorn et al., 2008; Sallee et al., 2009). Conventionally, MPH has stood as the mainstay of medication treatment of ADHD patients (Banaschewski et al., 2006). MPH is a reuptake inhibitor of catecholamines, including dopamine (DA) and noradrenaline (NA), which it does by blocking their transporters (Aron and Poldrack, 2005; Gatley et al., 1996). The affinity that MPH has with each catecholamine transporter is different: While the dissociation constant value, or K(i), of MPH to the NA transporter is 339 nM, that to the DA transporter is 34 nM (Bymaster et al., 2002). Thus, MPH is considered to have by far a greater effect on the DA system. Conversely, ATX, the first approved non-stimulant ADHD medication treatment, has been considered a selective NA reuptake inhibitor (Bolden-Watson and Richelson, 1993). The affinity that ATX has with these catecholamine transporters is biased toward the NA system with the K(i) of ATX to NA and DA transporters being 5 and 1451 nM, respectively (Bymaster et al., 2002).

These profiles demonstrate that both MPH and ATX act as monoamine agonists to normalize brain function in ADHD patients, but that they do so in different manners. ADHD is considered to include dysfunction of the DA and NA systems (Del Campo et al., 2011). In many ADHD neuroimaging studies, MPH has been shown to upregulate hypofunction in the DA system at the prefrontal cortex and the striatum, improving inhibitory functions (Del Campo et al., 2011; Epstein et al., 2007; Lewis et al., 2001; Rubia et al., 2011; Vaidya et al., 1998; Volkow et al., 2007) (Ellison-Wright et al., 2008; Frodl and Skokauskas, 2012; Nakao et al., 2011). On the other hand, it has been posited, based on findings from *in vitro* studies, that ATX acts on the NA system, mainly located in the locus coeruleus with axonal projections to the prefrontal and parietal cortices (Arnsten and Li, 2005; Del Campo et al., 2011; Seidman et al., 2005). However, there have not been any neuroimaging studies of the NA system in ADHD patients (Johnston et al., 2014).

Such a plausible functional difference might be reflected in differential neuropharmacological responses of ADHD children to MPH and ATX: there is a 30% non-responder rate for one or the other preferentially (Newcorn et al., 2008). Yet, the clinical therapeutic effects of these medications in ADHD children are not yet clearly understood. In addition, there is no evidenced-based method with objective markers for selecting effective medications. Furthermore, while these treatments have no symptomatic benefits in non-responders, their side effects remain present (Garnock-Jones and Keating, 2009). Even patients who do respond must be appropriately monitored to prevent possible side effects such as headaches, stomachaches, nausea, abdominal pain, decreased appetite and vomiting (Barkley, 2003; Garnock-Jones and Keating, 2009; Loeber et al., 1992; Milich et al., 2001; Murphy et al., 2001).

Preferably, the efficacy of either medication for ADHD children should be assessed both pre- and post-administration. One promising approach is the exploration of distinct biological markers and their testing with a non-invasive neuroimaging modality. A number of neuroimaging results for ADHD children (Beauregard and Levesque, 2006; Derefinko et al., 2008; Durston et al., 2003; Inoue et al., 2012; Ma et al., 2012; Monden et al., 2012a; Siniatchkin et al., 2012; Smith et al., 2006; Solanto et al., 2009; Vaidya et al., 1998), adolescents (Schulz et al., 2004; Tamm et al., 2004) and adults (Dibbets et al., 2009; Karch et al., 2010; Mulligan et al., 2011; Sebastian et al., 2012; Vasic et al., 2012) have shown that right middle and inferior frontal hypoactivation is distinctly associated with response inhibitory dysfunction. This gives rise to the possibility that activation in the inferior and middle frontal gyri could be a characteristic candidate as a neuropharmacological biomarker for ADHD (Aron and Poldrack, 2005). Indeed, a growing body of neuroimaging research has started to explore the neural basis for the clinical effectiveness of MPH in ADHD patients. An increasing number of fMRI-based neuropharmacological studies of MPH effects have demonstrated acute functional upregulation and normalization of the right middle and inferior frontal gyri after MPH administration (Epstein et al., 2007; Marquand et al., 2012; Vaidya et al., 1998).

Meanwhile, our previous fNIRS study (Monden et al., 2012b) assessed the pharmacological neuromodulation produced by MPH using a randomized, double-blind, placebo-controlled, crossover design. We reported that MPH normalized the hemodynamic responses in the right middle and inferior gyri during a motor-related inhibitory task (go/no-go task) using fNIRS on young ADHD children (Monden et al., 2012b), which was in accordance with previous evidence from a study with adult ADHD patients and fMRI (Morein-Zamir et al., 2014).

As demonstrated in our previous studies, fNIRS offers robust advantages such as its compactness (useful in confined experimental settings), affordable price, tolerance to body motion and accessibility (Ehlis et al., 2014; Herrmann et al., 2004; Herrmann et al., 2005; Hock et al., 1997; Matsuo et al., 2000; Matsuo et al., 2003; Miyai et al., 2001; Moriai-Izawa et al., 2012; Okamoto et al., 2004b; Okamoto et al., 2006; Shinba et al., 2004; Strangman et al., 2002a; Suto et al., 2004), which, in addition, have allowed it to be applied to the clinical assessment of ADHD children (Monden et al., 2012a; Monden et al., 2012b; Nagashima et al., 2014).

Conversely, it is often difficult to assess neuroactivation patterns during locomotor tasks with fMRI-based neuroimaging, and this can often cause problems in the neuro-functional assessment of school-aged ADHD children with hyperactivity. In fact, the rejection rate of fMRI studies is high: one study enrolling a relatively young sample of children (6 years and older) rejected 50% of ADHD subjects and 30% of normal control subjects (Durston et al., 2003). The high exclusion rate for ADHD patient populations in fMRI studies is mainly due to motion and lack of compliance (Yerys et al., 2009). According to the validation of our study and the fact that our drop rate has been 0% of a total 30 ADHD subjects (6–14 years old), our fNIRS-based examination is favorable in particular for measurements of active subjects, such as patients with ADHD, and should be further extended to neuropharmacological assessment of ATX effects in ADHD children.

Thus far, several fMRI studies on the effects of ATX have provided evidence of up-regulation of middle and inferior frontal gyrus activation in healthy control subjects (e.g., Graf et al., 2011; Hester et al., 2012), as with MPH. However, there are only three fMRI studies that have performed neuropharmacological assessments, utilizing double-blind, placebo-controlled designs, of the effects of ATX administration on inhibition function in ADHD patients including children (Cubillo et al., 2014; Schulz et al., 2012; Smith et al., 2013), and no fNIRS studies had been performed until now.

The lack of evidence associating a neuropharmacological mechanism with therapeutic improvement is tantamount to a missed opportunity for appreciating how ATX works, and such understanding is a vital step toward developing an objective, evidence-based neuropharmacological treatment for ADHD children. Thus we performed the current fNIRS study in order to assess acute neuropharmacological effects of ATX on inhibitory functions of ADHD children.

In the current study, we enrolled sixteen ADHD children and age- and sex-matched control subjects, and examined the neuropharmacological effects of ATX on inhibition control, utilizing a within-subject, double-blind, placebo-controlled design. We hypothesized that the ADHD subjects would exhibit hypoactivation in the right middle and inferior frontal gyri in comparison with control subjects, and that ATX would normalize hemodynamic responses during a go/no-go task while a placebo would not.

## 2 Material and methods

### 2.1 Subjects

Sixteen clinically referred, right-handed Japanese children with a mean age of 8.9 years (SD 2.2, range 6–14 years) who met the Diagnostic and Statistical Manual of Mental Disorders-IV (DSM-IV) criteria for ADHD participated in the study (Table 1). The Wechsler Intelligence Scale of Children — Third Edition (WISC-III) full IQ scores of subjects were all over 70 (mean 99.4, SD 14.4, range 75–126). Sixteen right-handed healthy control subjects were matched with the ADHD subjects according to age (mean 8.9, SD 2.2, range 6–13 years) and gender (14 boys and 2 girls). IQs of controls (mean 108.6, SD 8.1, range 92–121) were significantly (t = 2.4, p < 0.05) higher than those of ADHD subjects. All children and their parents gave oral consent for their participation in the study. Written consent was obtained from the parents of all subjects. The study was approved by the Ethics Committees of Jichi Medical University Hospital and the International University of Health and Welfare. The study was in accordance with the latest version of the Declaration of Helsinki. This study was registered to the University Hospital Medical Information Network Clinical Trials Registry (UMIN-CTR; 000007799) as “Monitoring of acute effects of ATX on cerebral hemodynamics in ADHD children: an exploratory fNIRS study using a go/no-go task”.

### 2.2 Experimental design

**Fig. 1:**
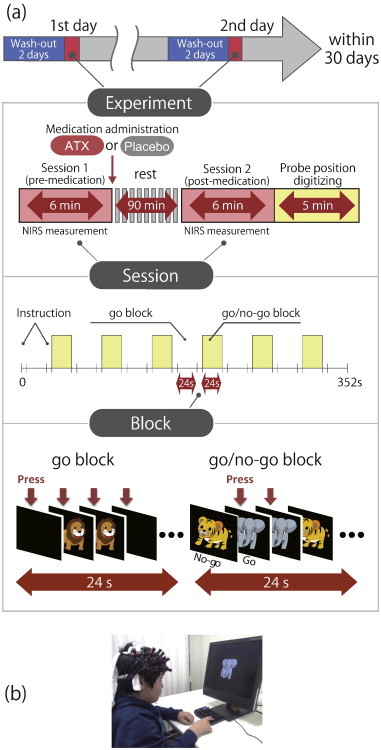
Experimental design. a) A schematic showing the flow of pre- and post-medication administration sessions for ADHD subjects. b) fNIRS measurements. Brain activity was measured while ADHD and control subjects performed the go/no-go task.

Fig. 1 summarizes the experimental procedure. We examined the effects of ATX in a randomized, double-blind, placebo-controlled, crossover study while the subjects performed a go/no-go task. We examined ADHD subjects twice (the times of day for both measurements were scheduled to be as close as possible), at least 2 days apart, but within 30 days. Control subjects only underwent a single, non-medicated session.

On each examination day, ADHD subjects underwent two sessions, one before drug (ATX or placebo) administration, and the other at 1.5 h after drug administration. Before each pre-administration session all ADHD subjects underwent a washout period of 2 days. We allowed subjects to take off the probe during waiting periods between the first and second sessions. Each session consisted of 6 block sets, each containing alternating go (baseline) and go/no-go (target) blocks. Each block lasted 24 s and was preceded by instructions displayed for 3 s, giving an overall block-set time of 54 s and a total session time of 6 min. In the go block, we presented subjects with a random sequence of two pictures and asked them to press a button for both pictures. In the go/no-go block, we presented subjects with a no-go picture 50% of the time, thus requiring subjects to respond to half the trials (go trials) and inhibit their response to the other half (no-go trials). Specifically, the instructions read in Japanese, “You should press the button as quickly as you can. Remember you want to be quick but also accurate, so do not go too fast.” Participants responded using the forefinger of the right hand. A go/no-go ratio of 50% was selected as it has been most often used in former neuroimaging studies (Dillo et al., 2010; Herrmann et al., 2005; Liddle et al., 2001; Menon et al., 2001; Vaidya et al., 1998). We presented pictures sequentially for 800 ms with an inter-stimulus interval of 200 ms during go and go/no-go blocks. At the beginning of each block, we displayed instructions (e.g., “press for giraffe or lion” for go conditions and “do not press for tiger” for go/no-go conditions) for 3 s to inform the subject about the new block. Each subject performed a practice block before any measurements to ensure their understanding of the instructions.

After ADHD subjects performed the first session, either ATX (Strattera) or a placebo was administered orally. The experimental design was as previously described (Monden et al., 2012a; Monden et al., 2012b). All patients were pre-medicated with ATX as part of their regular medication regimen. Specific, acute, experimental doses were the same as the patient's regular dose as described in Table 1.

### 2.3 Behavioral data analysis

We calculated the average reaction times (RT) for go trials, and accuracy rates for go and no-go trials in each go/no-go block for ADHD and control subjects. We averaged the accuracy and RTs across go/no-go blocks, and subjected the resulting values to statistical analyses as described in a subsequent section. We calculated mean RT for each participant by taking the average of RTs for correct go trials in the go/no-go block. We computed accuracy for go trials by dividing the number of correct responses (i.e., subjects pressed the button in go trials) by the total number of go trials for the go/no-go block. Similarly, we computed accuracy for no-go trials by dividing the number of correct inhibitions (i.e., subjects did not press the button in no-go trials) by the total number of no-go trials in the go/no-go block. We set the statistical threshold at 0.05 with the Bonferroni method for multiple-comparison error correction (i.e., significant: p < 0.05/2).

### 2.4 fNIRS measurement

We used the multichannel fNIRS system ETG-4000 (Hitachi Medical Corporation, Kashiwa, Japan), utilizing two wavelengths of near-infrared light (695 and 830 nm). We analyzed the optical data based on the modified Beer–Lambert Law (Cope et al., 1988) as previously described (Maki et al., 1995). This method enabled us to calculate signals reflecting the oxygenated hemoglobin (oxy-Hb), deoxygenated hemoglobin (deoxy-Hb), and total hemoglobin (total-Hb) signal changes, obtained in units of millimolar·millimeter (mM·mm) (Maki et al., 1995).

For statistical analyses, we focused on the oxy-Hb signal because of its higher sensitivity to changes in cerebral blood flow than that of deoxy-Hb and total-Hb signals (Hoshi, 2003; Hoshi et al., 2001; Strangman et al., 2002b), its higher signal-to-noise ratio (Strangman et al., 2002b), and its higher retest reliability (Plichta et al., 2006).

**Fig. 2:**
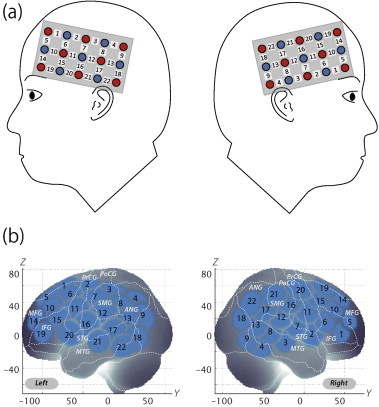
Spatial profiles of fNIRS channels. a) Left and right side views of the probe arrangements. fNIRS channel orientation is also illustrated. Detectors are shown as blue circles, illuminators as red circles, and channels as white squares. Corresponding channel numbers are indicated in black. b) Channel locations on the brain. Right- and left-side views are illustrated. Statistically estimated fNIRS channel locations (centers of blue circles) for control and ADHD subjects, and their spatial variability (SDs, radii of the blue circles) associated with the estimation are exhibited in MNI space.

We set the fNIRS probes so that they covered the lateral prefrontal cortices and inferior parietal lobe, referring to previous studies (Garavan et al., 1999; Herrmann et al., 2004; Herrmann et al., 2005; Liddle et al., 2001; Rubia et al., 2003). Specifically, we used two sets of 3 × 5 multichannel probe holders that consisted of eight illuminating and seven detecting probes arranged alternately at an inter-probe distance of 3 cm. This resulted in 22 channels (CH) per set. We defined the midpoint of a pair of illuminating and detecting probes as a channel location. We attached the bilateral probe holders in the following manner: (1) their upper anterior corners, where the left and right probe holders were connected by a belt, were symmetrically placed across the sagittal midline; (2) the lower anterior corners of the probe holder were placed over the supraorbital prominence; and (3) the lower edges of the probe holders were attached at the upper part of the auricles (Fig. 2). For spatial profiling of fNIRS data, we adopted virtual registration (Tsuzuki and Dan, 2014; Tsuzuki et al., 2007) for registering fNIRS data to MNI standard brain space (Brett et al., 2002). Briefly, this method enables us to place a virtual probe holder on the scalp based on a simulation of the holder's deformation and the registration of probes and channels onto reference brains in an MRI database (Okamoto et al., 2004a; Okamoto and Dan, 2005). Specifically, we measured the positions of channels and reference points, consisting of the Nz (nasion), Cz (midline central) and left and right preauricular points, with a 3D-digitizer in real-world (RW) space. We affine-transformed the RW reference points to the corresponding reference points in each entry in reference to the MRI database in MNI space. Adopting these same transformation parameters allowed us to obtain the MNI coordinates for the fNIRS channels and the most likely estimate of the locations of given channels for the group of subjects together with the spatial variability associated with the estimation (Singh and Dan, 2006). Finally, we estimated macroanatomical labels using a Matlab function that reads labeling information coded in a macroanatomical brain atlas, LBPA40 (Shattuck et al., 2008) and Brodmann's atlas (Rorden and Brett, 2000).

### 2.5 Analysis of fNIRS data

We preprocessed individual timeline data for the oxy-Hb and deoxy-Hb signals of each channel with a first-degree polynominal fitting and high-pass filter using cut-off frequencies of 0.01 Hz to remove baseline drift, and a 0.8 Hz low-pass filter to remove heartbeat pulsations. Note that Hb signals analyzed in the current study do not directly represent cortical Hb concentration changes, but contain an unknown optical path length that cannot be measured. Direct comparison of Hb signals among different channels and regions should be avoided as optical path length is known to vary among cortical regions (Katagiri et al., 2010). Hence, we performed statistical analyses in a channel-wise manner. From the preprocessed time series data, we computed channel-wise and subject-wise contrasts by calculating the inter-trial mean of differences between the peak Hb signals (4–24 s after go/no-go block onset) and baseline (14–24 s after go block onset) periods. For the six go/no-go blocks, we visually inspected the motion of the subjects and removed the blocks with sudden, obvious, discontinuous noise. We subjected the resulting contrasts to second-level, random-effects group analyses.

### 2.6 Statistical analysis

We statistically analyzed oxy-Hb signals in a channel-wise manner. Specifically, for control subjects, who were examined only once, we generated a target vs. baseline contrast for the session. For ADHD subjects, we generated the following contrasts: (1) pre-medication contrasts: the target vs. baseline contrasts for pre-medication conditions (either placebo or ATX administration) for the first day exclusively; (2) post-medication contrasts: the respective target vs. baseline contrasts for post-placebo and post-ATX conditions; (3) intra-medication contrasts: differences between post- and pre-medication contrasts for each medication (i.e., placebo^post-pre^ and ATX^post-pre^ contrasts); and (4) inter-medication contrasts: differences between ATX^post-pre^ and placebo^post-pre^ contrasts. To screen the channels involved in go/no-go tasks in normal control subjects, we performed paired t-tests (two-tails) on target vs. baseline contrasts. We set the statistical threshold at 0.05 with Bonferroni correction for family-wise errors. For thus-screened channels, we performed comparisons between control and ADHD for the following three ADHD contrasts: (1) pre-medication, (2) post-placebo, and (3) post-ATX. We performed independent two-sample t-tests (two-tails) on these contrasts with a statistical threshold of p < 0.05. To examine the medication effects on ADHD subjects, we performed paired t-tests (two-tails) with a statistical threshold of p < 0.05 for comparison between ATX^post-pre^ and placebo^post-pre^ (i.e., inter-medication contrast). We performed all statistical analyses with the PASW statistics (version 18 for Windows) (SPSS Inc., Chicago, USA) software package.

## 3 Results

### 3.1 Behavioral performance

The average accuracy for go and no-go trials and RT for correct go trials in the go/no-go block for control and ADHD subjects and ADHD inter-medication (placebo^post-pre^ vs. ATX^post-pre^) comparisons are summarized in Tables 2 and 3. We found no significant differences in accuracy for go and no-go trials or in RT for correct trials between control and pre-medication, post-placebo and post-ATX ADHD subjects (Table 2). The inter-medication contrast comparing the effect of ATX against the placebo revealed no significant differences in behavioral parameters between ADHD subjects (Table 3).

### 3.2 fNIRS analyses

**Fig. 3:**
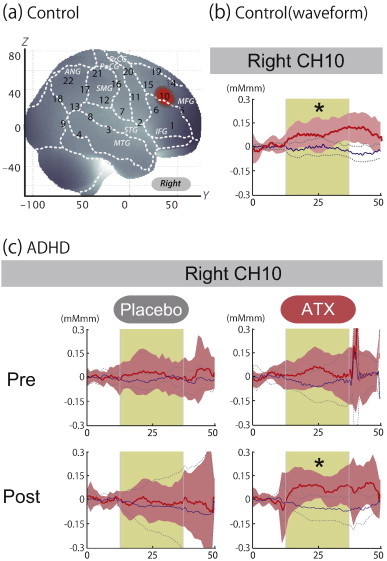
The waveforms of oxy-Hb (red line) and deoxy-Hb (blue line) signals. The beige area indicates the go/no-go task period. Significant (one-sample t-test, p < .05) conditions are indicated with asterisks. a) On-brain channel locations estimated for the group of subjects (including both ADHD and control) and exhibited in MNI space. The activated CH 10 located at the border of the right IFG and MFG is indicated in red. b) Grand averages for control subjects for CH 10 in the right hemisphere. Standard deviations among the 16 subjects are exhibited as pale red (oxy-Hb) and blue (deoxy-Hb) areas. Each timeline is adjusted to the average value for a baseline period of zero. Oxy-Hb and deoxy-Hb signals are shown in units of mM·mm. c) Grand averages for ADHD subjects. CH 10 on the right hemisphere for pre-/post- and placebo/ATX conditions.

First, we screened for any fNIRS channels involved in the go/no-go task for control and ADHD contrasts (pre-/post-placebo and pre-/post-ATX conditions; Fig. 3). We found a significant oxy-Hb increase in the right CH 10 (mean 0.095, SD 0.082, p < 0.05, Bonferroni-corrected, Cohen's d = 1.151) in control subjects. Conversely, in ADHD conditions, only post-ATX exhibited a significant oxy-Hb increase in the right CH 10 (mean 0.074, SD 0.063, p < 0.05, Bonferroni-corrected, Cohen's d = 1.165). Thus, we set the right CH 10 as a region-of-interest (ROI) for the rest of the study. This channel was located in the border region between the right MFG and IFG (MNI coordinates x, y, z (SD): 50, 37, 33 (16), MFG 68%, IFG 32%, Table 4) with reference to macroanatomical brain atlases (Rorden and Brett, 2000).

Comparison between oxy-Hb signals of the control and pre-medicated ADHD subjects revealed marginally significant activation of oxy-Hb signal in the right CH 10 in the control subjects (independent two-sample t-test, p < 0.1 Bonferroni-corrected, Cohen's d = 0.884; Table 2). This indicates that the control subjects exhibited higher right prefrontal activation during go/no-go tasks than did the pre-medicated ADHD children.

Then, we examined the effects of medication between control subjects and post-placebo-ADHD subjects, and between control subjects and post-ATX-ADHD subjects (Table 2). Oxy-Hb signal in control subjects was significantly higher than in post-placebo ADHD subjects (independent two-sample t-test, thresholded at p < 0.05 Bonferroni-corrected, Cohen's d = 1.176), while there was no significant difference between control subjects and post-ATX-ADHD subjects (independent two-sample t-test, thresholded at p = 0.430, Cohen's d = 0.283). This suggests that ATX administration normalized the impaired right prefrontal activation.

Finally, we examined whether there was an ATX-induced, but not placebo-induced, right prefrontal activation in ADHD subjects. In the inter-medication contrast, we found the right CH 10 to be significantly different between conditions (paired t-test, p < 0.05, Cohen's d = 0.663, Table 3). This result demonstrates that ATX, but not the placebo, induced an oxy-Hb signal increase during the go/no-go task.

### 3.3 Examination on the effects of IQ

Because we did not match the IQ of the ADHD and normal healthy control subjects, we additionally examined whether there was any possible effect of IQ. We performed correlation analyses for IQ and activation in the right CH 10 for ADHD subjects (ADHD post-placebo contrast) and control subjects, respectively. In ADHD subjects, Pearson's correlation coefficient was −0.043 (p = 0.871), while that in control subjects was −0.023 (p = 0.934): In neither analysis did we find any significant correlation with a meaningful effect size. Further, we compared the two correlation coefficients, but did not find any significant difference (Fischer's z = 0.056, p = 0.956). This led us to conclude that there was no correlation between IQ and the activation in the right CH 10 in either group.

## 4 Discussion

### 4.1 Overview

Our current study, using a double-blind, placebo-controlled, crossover design, provided the first fNIRS-based neuropharmacological evidence of the acute ATX effect on inhibitory control in school-aged ADHD children. Through assessing cortical activation data of ADHD and healthy control subjects performing a go/no-go task reflecting function of the motor-related inhibitory network, we revealed that the right IFG/MFG is a neural substrate of ATX effects in ADHD children based on the following findings. First, ADHD children exhibited reduced cortical activation in the right IFG/MFG during go/no-go task blocks compared to control subjects. Second, the reduction of right IFG/MFG activation was acutely normalized after ATX administration in ADHD children. Third, the ATX-induced right IFG/MFG activation was significantly greater than placebo-induced activation during go/no-go task blocks.

The recovered right IFG/MFG activation in ADHD children detected by fNIRS measurements after ATX administration is consistent with our previous studies using MPH (Monden et al., 2012a,b). These results suggest that normalized right IFG/MFG activation during a go/no-go task, as observed using fNIRS, may serve as a robust neurobiological marker for evaluating ATX effects on ADHD children as with evaluating MPH effects.

### 4.2 Behavioral performance for go/no-go task

One of the most commonly used experimental paradigms for evaluating response inhibition is the go/no-go task, in which subjects are generally required to inhibit a prepotent response when no-go stimuli are presented within a sequence of go stimuli (Simmonds et al., 2008). This is an essential cognitive function required in daily life, and impaired response inhibition is a potential biomarker candidate for ADHD in children (Barkley, 1997). Because of this, a number of go/no-go paradigms have been widely adopted to explore the disinhibitory nature of ADHD in fMRI studies (Aron and Poldrack, 2005; Nigg, 2000).

In general, a go/no-go task allows the assessment of detailed aspects of inhibitory response controls reflected in a variety of parameters (Newcorn et al., 2001): Errors of omission (the absence of response to a standard stimuli) are generally interpreted as a symptom of inattention; errors of commission and overly reduced reaction times with standard stimuli are commonly considered indicators of impulsivity (Newcorn et al., 2001). However, our current study did not show any significant differences in behavioral performance between ADHD children and control subjects. Thus far, we have observed inconsistency in behavioral data for ADHD children: our previous studies (Monden et al., 2012b) showed performance impairment in ADHD children compared with control subjects. However, our fNIRS studies have consistently exhibited hypoactivation in the MFG/IFG in pre-medicated ADHD children without corresponding behavioral effects. This tendency is reminiscent of an fMRI study by Smith et al. (2006) reporting that the go/no-go task parameters showed no difference between ADHD children and IQ- and age-matched healthy controls, while hypoactivation in the bilateral prefrontal and right parietal lobes was found in the ADHD patients.

These inconsistencies among the results of both studies represent the difficulty in interpreting behavioral parameters compared with brain activation patterns for detecting cognitive dysfunction in ADHD children.

### 4.3 fNIRS examination of go/no-go task and ATX effects

In our current study, we detected brain activation in the right MFG/IFG during go/no-go task blocks in the healthy control subjects. This activation pattern is in accord with that found in previous fMRI studies, and this region is regarded as especially important for inhibitory control (Aron and Poldrack, 2005; Morein-Zamir et al., 2014). This led us to conclude that our current fNIRS measurements robustly extracted concurrent activations for response inhibition in the right prefrontal cortex in control subjects.

In ADHD conditions, ATX-induced normalization in the MFG/IFG, as identified using fNIRS, is consistent with former MPH-related studies (Monden et al., 2012b). Also, these activation patterns are similar to the results of previous fMRI studies (Cubillo et al., 2014; Schulz et al., 2012).

In a different vein of studies using animals, both ATX and MPH led to increased NA and DA in the prefrontal cortex of mice (Koda et al., 2010) and rats (Ago et al., 2014). Taken together, it would be natural to conclude that administration of either ATX or MPH increases NA and DA concentration in the prefrontal cortex, leading to normalization of inhibitory control in ADHD children. However, this does not necessarily suggest that both medications affect prefrontal functions via the same neuropharmacological mechanism. We must note here that ATX and MPH have an almost opposite affinity to DA and NA transporters. While MPH has a 10-fold higher affinity to DA than to NA transporters, ATX has a 300-fold higher affinity to NA than to DA transporters (Bymaster et al., 2002).

According to this evidence, we speculate that MPH has by far larger effects on the DA system between the prefrontal and striatal regions, while ATX has far larger effects on the locus coeruleus NA system between the prefrontal and coeruleus areas (Singh-Curry and Husain, 2009). Thus, what appears as the similar activation patterns induced by ATX and MPH in the prefrontal cortex may reflect different neural substrates. In order to elucidate the precise neuropharmacological mechanism underlying the right prefrontal functional normalization by ATX and MPH, further investigation is necessary.

### 4.4 Clinical implications

In the present study, we selected a go/no-go task paradigm with alternating go blocks as baseline blocks and go/no-go blocks as target blocks without rest segments in between active (go and go/no-go) task blocks. Tsujii et al. (2011) and Cui et al. (2011) also adopted a similar block designed for go/no-go tasks, and treated the go task period as the baseline for contrast with the go/no-go task period when analyzing fNIRS signals. This paradigm was set primarily because of the difficulty with ADHD patients staying still without performing any tasks, which may lead to unexpected movements or hyperactive behavior. In addition, we omitted rest blocks to save time, as a long experiment time would bore ADHD subjects. Furthermore, the go and go/no-go block design is commonly used in fMRI studies (Altshuler et al., 2005; Dillo et al., 2010; Ma et al., 2012; Vaidya et al., 1998). Thus, considering comparisons across modalities, the use of the go/no-go task paradigm in the current study is appropriate.

Another merit of the block-design paradigm is that the baseline blocks serve as a motor control for the target blocks. Schecklmann et al. (2008) used a weekday-reciting task as a baseline block and a word fluency task as a target block, and used fNIRS to analyze the difference in signal between the two tasks. In this paradigm, movement and muscle artifacts in the task condition are expected to be neutralized with the use of a control condition with a similar motor output. Similarly, we adopted the go task as the baseline task. As the physical movements made by children during the go task are similar to those of the go/no-go task, movement and muscle artifacts are expected to be ruled out. Accordingly, activation during the go/no-go task block is considered to reflect inhibitory control; thus, this paradigm is more appropriate than one using a rest block as the baseline. Although fNIRS studies often use a paradigm where rest and task blocks are alternately performed (Herrmann et al., 2005), we suggest that it would be more applicable for studies involving younger ADHD children to adopt the alternating go and go/no-go block design.

Reminiscent of our study demonstrating the clinical utility of fNIRS-based assessment of the efficacy of an acute single dose of MPH to ADHD children, here ATX has been shown to be similarly effective: the current study demonstrates the utility of fNIRS-based assessment of the efficacy of an acute single dose of ATX administered to ADHD children. fNIRS-based assessment has a fundamental clinical importance as a diagnostic tool and for therapeutic encouragement. For the diagnostic aspect, we demonstrated that fNIRS-based measurement can reveal the effects of an acute single dose of ATX with higher sensitivity than can behavioral parameters. The moderately large effect size of the acute single dose of ATX as compared to that of the placebo (Cohen's d = 0.663) demonstrates that fNIRS-based assessment can serve as a comparably effective diagnostic tool for the effect of ATX in ADHD children, especially those at elementary-school ages.

Moreover, fNIRS-based measurement could provide therapeutic encouragement to ADHD children and their families. One major problem of medication treatment, which is common with both AXT and MPH, is the high discontinuation rate estimated at between 36 and 85% (Adler and Nierenberg, 2010; Habel et al., 2005). Since guardians' subjective feelings about the efficacy of medication stand as a major cause for the discontinuation of medication treatment with ADHD children (Toomey et al., 2012), encouragement of family members of ADHD children by demonstrating therapeutic success may facilitate successful ATX treatment. Objective demonstration of ATX effects as visualized with cortical activation observed with fNIRS-based measurements could act as an informative guide, encouraging ADHD children and their guardians to continue ATX treatment.

### 4.5 Limitations

As discussed above, the current study has demonstrated the ATX-effect assessment on inhibitory control in ADHD children using fNIRS. However, for adequate understanding of current findings, several issues need to be addressed.

First, IQs of control children (mean 108.6, SD 8.1, range 92–121) were significantly (t = 2.4, p < 0.05) higher than those of ADHD children (mean 99.4, SD 14.4, range 75–126). IQ has been reported as having a negative correlation with ADHD scores (Goodman et al., 1995). Since IQ is not independent of ADHD, IQ matching to control subjects could remove a disorder-related variance from the ADHD group (Miller and Chapman, 2001). Further study with a larger sample size may have to be performed in order to explore the possible effects of IQ.

The second limitation of this study is that controls were only tested once, while children with ADHD were tested a total of four times. The practice effect of multiple testing in ADHD children was controlled for by the counterbalanced design. Ethical limitations prevented us from testing healthy controls under stimulant medication, as well as from having them wait for 90 min to retest; however, we need to explore ways to eliminate potential training effects with appropriate experimental procedures. Since there are no studies on assessing order and learning effects of go/no-go tasks associated with fNIRS signals, this would be an interesting and essential area for future study.

## 5 Conclusion

The current study examining the effects of a single acute dose of ATX on inhibitory control in ADHD children using a double-blind, placebo-controlled, crossover design, revealed the following findings. First, the activation foci (right IFG/MFG), which are involved in inhibition control, were activated in control subjects performing a go/no-go task, but not in ADHD children. Second, the ATX-induced right IFG/MFG activation was significantly greater than placebo-induced activation during go/no-go task blocks. Third, the activation in the right IFG/MFG region was normalized after ATX administration. Taken together, these findings led us to conclude that the activation in the MFG/IFG could provide an objective neuro-functional biomarker that indicates the effects of ATX on inhibitory control in ADHD children. This fNIRS-based examination on the effect of ATX is applicable to ADHD children at elementary school ages including those as young as 6 years old. Thus, we believe that fNIRS-based examination is a promising clinical tool that could enable the early diagnosis and treatment of ADHD children.

## Figures and Tables

**Supplementary Figure:**
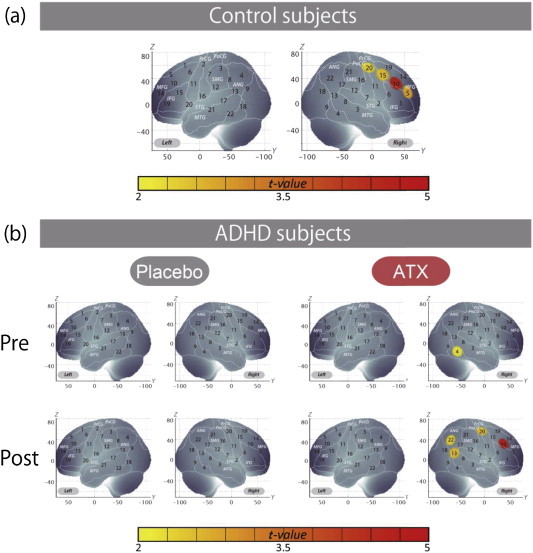
Cortical activation patterns of control subjects (a) and of ADHD subjects (b) shown as t-maps of oxy-Hb signal, with significant t-values (one-sample *t*-test, *p* < .05 uncorrected) being shown according to the color bar. We screened for any channels involved in the go/no-go task for the control subjects. A significant increase was found in four channels on the right hemisphere (CH 5, 10, 15 and 20). Among these channels, the right CH 10 exhibited the most significant activation and was the only channel remaining after family-wise error correction (mean 0.095, SD 0.082, p < 0.05, Bonferroni-corrected, Cohen's d = 1.151). Therefore, the right CH 10 was determined as a channel of interest in the main analyses. In ADHD subjects, significant ATX effects on oxy-Hb increase were found in four channels on the right hemisphere (CH 10, 13, 20, and 22). We found that no channels were activated in the pre-placebo condition, but that the right inferior and middle temporal gyri (CH 4, p < 0.05, uncorrected) were activated in the pre-ATX condition. In addition, there were no significant activations in the post-placebo condition, while significant activation was found in the right prefrontal and parietal regions in the post-ATX condition (CH 10, 13, 20 and 22, p < 0.05, uncorrected). The marginal activations in the right inferior and middle temporal gyri in the pre-ATX condition might reflect alternative or compensatory activations specific to ADHD children, but further studies are necessary to examine the reproducibility and underlying functional mechanisms of these results.

**Table 1:** Demographic and clinical profiles for ADHD subjects.

ID	Age (years)	Sex	ADHD subtype	Complication	ATX (mg)	WISC-III Full IQ	Duration of ATX exposure (months)	Other medications	1st day	2nd day
1	10	M	Combined	none	50	109	27	None	Placebo	ATX
2	7	M	Combined	none	35	118	2	None	ATX	Placebo
3	14	M	Combined	ASD	35	90	7	None	ATX	Placebo
4	10	M	Combined	ASD	40	95	7	None	Placebo	ATX
5	6	M	Combined	ASD	25	84	4	None	ATX	Placebo
6	8	M	Inattentive	ASD	20	126	6	None	Placebo	ATX
7	9	M	Inattentive	ASD	40	110	10	None	Placebo	ATX
8	10	M	Inattentive	ASD	10	82	24	None	ATX	Placebo
9	8	M	Combined	none	15	92	3	None	ATX	Placebo
10	6	M	Combined	ASD	5	75	6	None	Placebo	ATX
11	11	F	Combined	ASD	15	85	4	Valproic acid	Placebo	ATX
12	8	M	Inattentive	ASD	10	95	3	None	ATX	Placebo
13	12	M	Combined	ASD	25	114	18	None	ATX	Placebo
14	8	M	Combined	ASD	5	107	12	None	ATX	Placebo
15	9	F	Inattentive	ASD	25	101	22	None	Placebo	ATX
16	6	M	Combined	ASD	15	107	6	None	Placebo	ATX
Mean	8.8				23.1	99.3	10			
SD	2.2				13.6	14.4	8			

Abbreviations: SD, standard deviation; ASD, autism spectrum disorders.

**Table 2:** Go/no-go task performance and functional data for control and ADHD subjects.

	Control		ADHD														
			Pre-medication (mean of pre-placebo and ATX)					Post-placebo vs. control					Post-ATX vs. control				
	Mean	SD	Mean	SD	t	p		Mean	SD	t	p		Mean	SD	t	p	
*Performance data*																	
RT for correct trials (ms)	426.3	59.4	435.0	50.8	0.444	0.660	n.s.	435.7	67.7	0.331	0.746	n.s.	429.2	56.8	0.116	0.910	n.s.
Accuracy for go trials (%)	96.6	6.0	97.8	3.6	0.711	0.483	n.s.	96.7	3.6	0.061	0.953	n.s.	97.7	4.3	0.614	0.549	n.s.
Accuracy for no-go trials (%)	95.3	5.7	94.4	3.2	0.554	0.584	n.s.	93.7	6.3	0.936	0.364	n.s.	93.9	5.0	0.834	0.417	n.s.

*Functional data*																	
Oxy-Hb right CH 10 (mM·mm)	0.095	0.083	0.025	0.077	2.617	0.019	†	−0.016	0.105	3.326	0.002	**	0.074	0.063	0.800	0.430	n.s.

Performance data (RT for correct trials and accuracy rates for go and no-go trials) is presented for go/no-go blocks. Oxy-Hb data includes right CH 10. For ADHD subjects, data for post-medication with placebo and ATX are shown. t-Values, p-values and statistical significances were the results of t-tests between control and each ADHD condition. Abbreviations: SD, standard deviation; t, t-value; p, p-value. Statistical significances are presented as follows: †, p < 0.10 Bonferroni-corrected; **, p < 0.01 Bonferroni-corrected; and n.s., not signiﬁcant.

**Table 3:** ADHD inter-medication (ATX^post-pre^ vs. PLA^post-pre^) comparison.

	ATX^post-pre^ minus PLA^post-pre^		ATX^post-pre^ vs. PLA^post-pre^		
	Mean	SD	t	p	
*Performance data*					
RT for correct trials (ms)	13.8	61.2	0.902	0.381	n.s.
Accuracy for go trials (%)	1.2	4.7	1.002	0.332	n.s.
Accuracy for No-go trials (%)	−0.7	6.5	−0.401	0.694	n.s.

*Functional data*					
Oxy-Hb right CH 10 (mM·mm)	0.074	0.112	2.655	0.018	**

Performance data (RT for correct trials and accuracy rates for go and no-go trials) is presented for go/no-go blocks. Data for inter-medication comparisons (i.e., ATX^post-pre^ vs. PLA^post-pre^) are shown for ADHD subjects. Mean values were calculated by first subtracting the values of ATX^post-pre^ from those of PLA^post-pre^ for each subject and then averaging the resulting values across subjects. SD were similarly calculated. t-Values, p-values, and statistical significance were the results of two-sample t-tests between ATX^post-pre^ and PLA^post-pre^. Abbreviations: ATX^post-pre^, the difference between post- and pre-ATX; PLA^post-pre^, the difference between post- and pre-PLA; SD, standard deviation; t, t-value; p, p-value. Statistical signiﬁcances are as follows: *, p < 0.05; **, p < 0.01; and ns, not signiﬁcant.

**Table 4:** Spatial profiles of the channels screened for involvement with go–no-go tasks.

	MNI coordinates					
	x, y, z (SD)	Macroanatomy	Prob	Brodmann area		Prob
CH 10	50, 37, 33 (16)	R middle frontal gyrus	.68	45	Pars triangularis Broca's area	.61
		R inferior frontal gyrus	.32	46	Dorsolateral prefrontal cortex	.15
				44	Pars opercularis, part of Broca's area	.06
				9	Dorsolateral prefrontal cortex	.01

Abbreviations: Prob, probability; SD, standard deviation; R, right.
